# Supplementary material for: Remote Assessment of Parkinson’s Disease Patients Amidst the COVID-19 Lockdown in Mexico
Source: Brain Sci. 2023 Jul 22;13(7):1114. doi: 10.3390/brainsci13071114 (PMC10377101; doi:10.3390/brainsci13071114)
Supplement: Supplementary file 1 [file brainsci-13-01114-s001.zip › brainsci-2478693-supplementary.pdf]

Table S1. Patient questionnaire.

**1. How many virtual visits did you have?**<br>*Answer: [Quantity]*	**O**
**2. What device did you use to carry out the virtual visit(s)?**<br>*a. Desktop*<br>*b. Smartphone*<br>*c. Laptop*<br>*d. Tablet / iPad*	**O**
**3. I found setting up for the visit understandable and easy.**<br>*10-point Likert Scale (1= highly disagree, 10= highly agree)*	**S/P**
**4. How long did you spend setting up for your telemedicine visit (in minutes)? This would include any time you spent preparing your computer hardware or software or internet services prior to the visit.**<br>*Answer: [Quantity]*	**S/P**
**5. How long did you spend on an in-person consultation (in minutes)? This would include travel time from home to the clinic and time in the waiting room before you see the doctor.**<br>*Answer: [Quantity]*	**O**
**6. How do you feel the time commitment for your telemedicine visit compared to a face-to-face visit with your neurologist?**<br>*10-point Likert Scale (1= much more time, 10= much less time)*	**S**
**7. The clinical care I received during the telemedicine visit was ______ than a face-to-face visit.**<br>*10-point Likert Scale (1= much worse, 10= much better)*	**S**
**8. I felt I had the doctor's full attention during the telemedicine visit.**<br>*10-point Likert Scale (1= highly disagree, 10= highly agree)*	
**9. I felt my doctor could see and hear me well.**<br>*10-point Likert Scale (1= highly disagree, 10= highly agree)*	**QS**
**10. I felt I could see and hear my doctor well.**<br>*10-point Likert Scale (1= highly disagree, 10= highly agree)*	**QS**
**11. I felt my body was safe during the telemedicine exam.**<br>*10-point Likert Scale (1= highly disagree, 10= highly agree)*	**QS**
**12. I felt my privacy was safe during the telemedicine visit.**<br>*10-point Likert Scale (1= highly disagree, 10= highly agree)*	**QS**
**13. Were you able to establish a personal connection to your specialist during the visit? How does it compare to an in-person visit?**<br>*a. Yes, I established more of a personal connection with an in-person visit.*<br>*b. Yes, I established the same level of personal connection as with an in-person visit.*<br>*c. Yes, but I established less of a personal connection than with an in-person visit.*<br>*d. No, I did not establish a personal connection to my specialist during the visit.*	**S**
**14. How satisfied were you with the specialist's ability to provide recommendations to improve your quality of life?**<br>*10-point Likert Scale (1= very unsatisfied, 10= very satisfied)*	**S**

**15. How clear were the instructions the doctor gave me regarding changes in my drug treatment?**<br>*10-point Likert Scale (1= not clear at all, 10= very clear)*	**S**
**16. Do you feel that a service like this, which would allow patients to receive care from a specialist via web-based videoconferencing, would be valuable?**<br>*a. Yes*<br>*b. No*	**S**
**17. I am pleased with the outcome of my telemedicine visit.**<br>*10-point Likert Scale (1= highly disagree, 10= highly agree)*	**S**
**18. If given the option, I would like my future appointments by telemedicine with my neurologist whenever feasible.**<br>*10-point Likert Scale (1= highly disagree, 10= highly agree)*	**S**
**19. How likely are you to recommend telemedicine to another patient with the same or similar health condition?**<br>*10-point Likert Scale (1= very unlikely, 10= very likely)*	**S**
**20. If you wish to add any comment (positive or negative) in relation to the telemedicine visits, you can write it below.**<br>*Free answer.*	**O**

S, satisfaction-related items; S/P, set-up/preparation-related items; QS, quality of service-related items; O, other.
